# CMV-associated T cell and NK cell terminal differentiation does not affect immunogenicity of ChAdOx1 vaccination

**DOI:** 10.1172/jci.insight.154187

**Published:** 2022-03-22

**Authors:** Hannah R. Sharpe, Nicholas M. Provine, Georgina S. Bowyer, Pedro Moreira Folegatti, Sandra Belij-Rammerstorfer, Amy Flaxman, Rebecca Makinson, Adrian V.S. Hill, Katie J. Ewer, Andrew J. Pollard, Paul Klenerman, Sarah Gilbert, Teresa Lambe

**Affiliations:** 1 Jenner Institute and; 2 Translational Gastroenterology Unit, Experimental Medicine Division, Nuffield Department of Medicine, University of Oxford, United Kingdom.; 3 Oxford Vaccine Group, Department of Paediatrics, Medical Sciences Division, University of Oxford and the National Institute for Health Research (NIHR) Oxford Biomedical Research Centre, Oxford, United Kingdom.; 4 Chinese Academy of Medical Science (CAMS) Oxford Institute (COI), University of Oxford, Oxford, United Kingdom.

**Keywords:** COVID-19, Vaccines, NK cells, T cells

## Abstract

Cytomegalovirus (CMV) is a globally ubiquitous pathogen with a seroprevalence of approximately 50% in the United Kingdom. CMV infection induces expansion of immunosenescent T cell and NK cell populations, with these cells demonstrating lower responsiveness to activation and reduced functionality upon infection and vaccination. In this study, we found that CMV^+^ participants had normal T cell responses after a single-dose or homologous vaccination with the viral vector chimpanzee adenovirus developed by the University of Oxford (ChAdOx1). CMV seropositivity was associated with reduced induction of IFN-γ–secreting T cells in a ChAd-Modified Vaccinia Ankara (ChAd-MVA) viral vector vaccination trial. Analysis of participants receiving a single dose of ChAdOx1 demonstrated that T cells from CMV^+^ donors had a more terminally differentiated profile of CD57^+^PD1^+^CD4^+^ T cells and CD8^+^ T cells expressing less IL-2Rα (CD25) and fewer polyfunctional CD4^+^ T cells 14 days after vaccination. NK cells from CMV-seropositive individuals also had a reduced activation profile. Overall, our data suggest that although CMV infection enhances immunosenescence of T and NK populations, it does not affect antigen-specific T cell IFN-γ secretion or antibody IgG production after vaccination with the current ChAdOx1 nCoV-19 vaccination regimen, which has important implications given the widespread use of this vaccine, particularly in low- and middle-income countries with high CMV seroprevalence.

## Graphical abstract

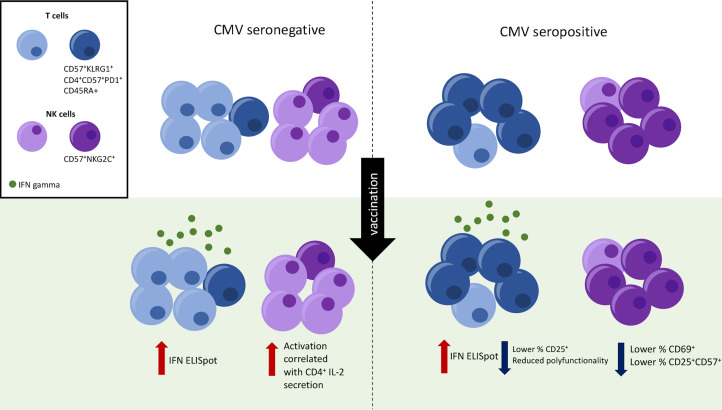

## Introduction

Cytomegalovirus (CMV) infection establishes latency within the host, thus ingraining a unique immunosenescent phenotype on the immune system (1). This includes expansion of terminally differentiated T cells expressing CD57 and KLRG1 and lacking activation markers, including CD27 and CD28, and NK cells with an expanded profile of CD57 and NKG2C type II integral membrane protein (CD159c, NKG2C) double positive. In Europe, seropositivity ranges between approximately 30% and 60% of the adult population (2). By contrast, 85% of children in sub-Saharan Africa are infected by the time they are 1 year old (3, 4), Globally, CMV seropositivity also increases with age (5).

Recently, the importance of understanding the role of CMV infection in vaccine immunogenicity has been highlighted in research conducted in both younger and older adults. In some studies, CMV seropositivity correlates with overall lower vaccine-specific T cell IFN-γ secretion and an increase in background levels of inflammatory granzyme B, IL-6, or TNF (6–14). Other research demonstrates that humoral responses are also reduced in CMV-seropositive older adults vaccinated against influenza (10, 11, 15). Furthermore, NK cell functionality and cytotoxicity are reduced in some vaccinated individuals with CMV (16–19). In contrast, other research has shown increased humoral responses to influenza vaccination in adults with CMV (20–23) or no discernible correlation between CMV serostatus and vaccine response (8, 9, 24, 25).

Replication-deficient viral-vectored vaccines include chimpanzee adenovirus–vectored (ChAd-vectored) vaccines, e.g., ChAd developed by the University of Oxford (ChAdOx1), ChAd serotype 3 (ChAd3), and the poxvirus-derived vector Modified Vaccinia Ankara (MVA). Viral-vectored vaccines have been tested in clinical trials against multiple pathogens and have demonstrated significant cellular and humoral immunogenicity of the gene-insert product after a single vaccine dose (26–28) and when administered in prime-boost regimens (29–34).

In this paper, we investigated the effect of CMV infection on the immunogenicity of single-dose and prime-boost ChAdOx1 and MVA viral vector vaccines across 3 clinical trials against outbreak or pandemic pathogens. There was no significant loss of cellular cytotoxicity or antibody titers in single-dose or homologous prime-boost ChAdOx1 vaccination, which is of importance considering the widespread use of ChAdOx1 nCoV-19 in countries with high rates of CMV seropositivity. However, antigen-specific T cell IFN-γ was lower following ChAdOx1-MVA prime-boost vaccination. Analysis of the single-dose ChAdOx1 cohort revealed a reduced activation profile and more terminal differentiation in T cells and NK cells from CMV^+^ participants. Overall, these data demonstrate that T cell IFN-γ and antibody production following single-dose and homologous prime-boost ChAdOx1 vaccination is not affected by CMV serostatus in young UK adults.

## Results

### Vaccine trials and CMV serotyping.

This study includes an analysis of a trial group receiving single-dose vaccination with the SARS-CoV-2 vaccine ChAdOx1 nCoV-19 and a trial group receiving a homologous prime-boost ChAdOx1 nCoV-19 vaccination regimen after 28 days. The ChAdOx1 nCoV-19 (AZD1222) vaccine encodes the spike glycoprotein from the original isolate of SARS-CoV-2. Young adult participants (*n* = 44 aged 18–55) received a single dose of 5 × 10^10^ viral particles (v.p.) ChAdOx1 nCoV-19 (group ChAdOx1 S-D). A separate group of 27 participants (aged 18–55) received a homologous 5 × 10^10^ v.p. boost dose of ChAdOx1 nCoV-19 at an interval of 28 days (group ChAdOx1 P-B; Table 1).

We also analyzed 2 vaccine trials with heterologous viral vector vaccination for this study. Young adults (*n* = 16, aged 18–50) received a prime-boost regimen of 3.6 × 10^10^ v.p. ChAd3 Ebola virus-glycoprotein (EBOV-GP) and 1 × 10^8^ PFU MVA EBOV-GP expressing the glycoprotein from the Zaire Ebola virus strain Mayinga (EBO-Z) administered at an interval of 7 days (group ChAd3-MVA) (31). The third trial included young adults aged 18–55 who were administered a prime-boost vaccination regimen of 2.5 × 10^10^ v.p. ChAdOx1 and 2.5 × 10^8^ PFU MVA expressing the nucleoprotein (NP) and matrix 1 (M1) fusion protein from influenza A (group ChAdOx1-MVA) administered at an interval of 8 weeks (group 1) or 52 weeks (group 2; Table 1). All trials analyzed were conducted in the United Kingdom, and immunogenicity reports have been published previously (27, 29, 31, 35, 36).

Trial participants were screened for CMV using a commercially available anti-CMV IgG ELISA kit. The ChAdOx1 S-D group had a CMV seropositivity of 29.5% (13/44), and the ChAdOx1 P-B group had a seropositivity of 46.6% (20/49). In the ChAdOx1-MVA vaccine cohort, 26% (6/23) of donors were CMV seropositive. Previous analysis of the ChAd3-MVA cohort demonstrated seroprevalence of cytomegalovirus in 50% of donors from the United Kingdom (8/16) (37).

### Expansion of terminally differentiated T cell populations with reduced activation in CMV-seropositive trial participants.

**Figure 1:**
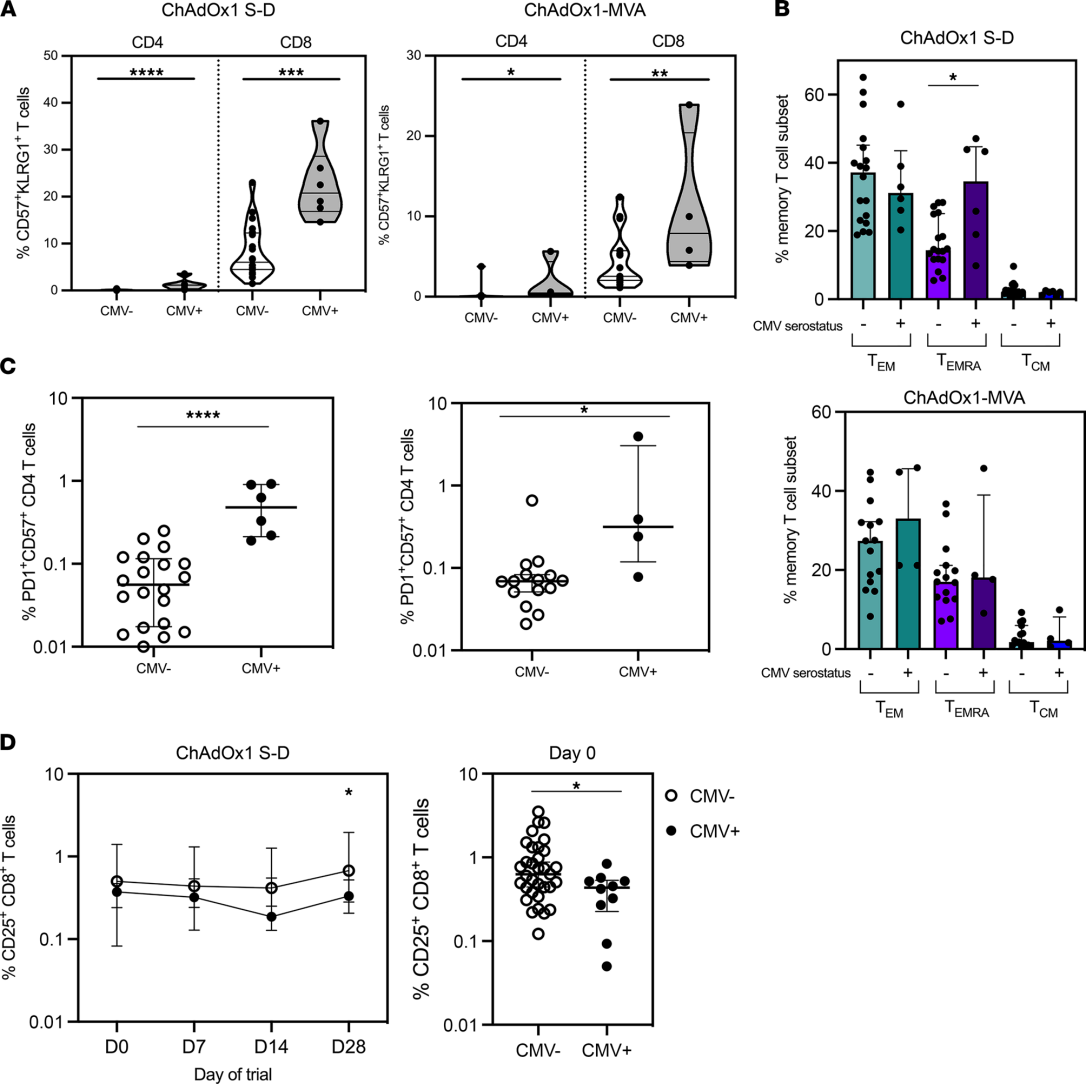
T cell phenotype of trial participants when stratified by CMV serostatus. (**A**) CD57^+^KLRG1^+^ CD4^+^ and CD8^+^ T cells in CMV^+^ and CMV^–^ donors from ChAdOx1 S-D and ChAdOx1-MVA trial cohorts. (**B**) CD45RA/CCR7 memory profile of CD8^+^ T cells. (**C**) CD4^+^CD57^+^PD1^+^ T cells in CMV^+^ and CMV^–^ individuals. (**D**) CD25 expression on CD8^+^ T cells stratified by CMV serostatus and pooled day 0 CD8^+^CD25^+^ T cells. ChAdOx1 S-D: *n* = 20 CMV seronegative, *n* = 6 CMV seropositive. ChAdOx1-MVA: *n* = 15 CMV seronegative, *n* = 4 CMV seropositive. Open circles = CMV seronegative, closed circles = CMV seropositive. Statistics conducted using Mann-Whitney *U* test and mixed effects analysis with Holm-Šidák multiple comparisons. *P < 0.05, **P < 0.01, ***P < 0.005, ****P < 0.0001. Error bars shown as median ± IQR.

We have previously shown within the heterologous ChAd3-MVA vaccine cohort that CMV-seropositive participants had a terminally differentiated T cell phenotype with an expanded population of CD57^+^KLRG1^+^ T cells and reduced expression of CD27 and CD28 (37). A cohort of participants from the ChAdOx1 S-D group (*n* = 26, CMV seropositive = 6) and the ChAdOx1-MVA group (*n* = 19, CMV seropositive = 4) were selected for phenotyping by flow cytometry. We demonstrate here that CD4^+^ and CD8^+^ T cells from CMV-seropositive donors in both the ChAdOx1 S-D cohort and the ChAdOx1-MVA cohort have an expanded population expressing CD57 and KLRG1 at day 0 prevaccination (ChAdOx1 S-D, *P* < 0.0003; ChAdOx1-MVA, *P* < 0.048; Figure 1A). Within the CD45RA/CCR7 T cell memory compartment of the ChAdOx1 S-D cohort, CD8^+^ T cells from CMV-seropositive donors had an expanded CD45RA^+^CCR7^–^ effector memory RA T cell (T_EMRA_) population (*P* = 0.038, day 0), although this was not observed in the ChAdOx1-MVA cohort (Figure 1B). An expansion of terminally differentiated memory T cells is consistent with our previous report describing a higher frequency of CD8^+^CD45RA^+^ T_EMRA_ cells in CMV-seropositive participants from the ChAd3-MVA trial cohort (37). We also observed significantly more CD57^+^PD1^+^CD4^+^ T cells in CMV-seropositive participants in both trials at day 0 (ChAdOx1 S-D, *P* < 0.0001; ChAdOx1-MVA, *P* = 0.0196; Figure 1C) and CD8^+^ T cells with reduced expression of CD25 across measured time points (ChAdOx1 S-D, day 28, *P* = 0.035; pooled ChAdOx1 S-D and ChAdOx1-MVA, day 0, *P* = 0.04; Figure 1D).

### CMV infection reduces T cell immunogenicity following heterologous prime-boost vaccination.

**Figure 2:**
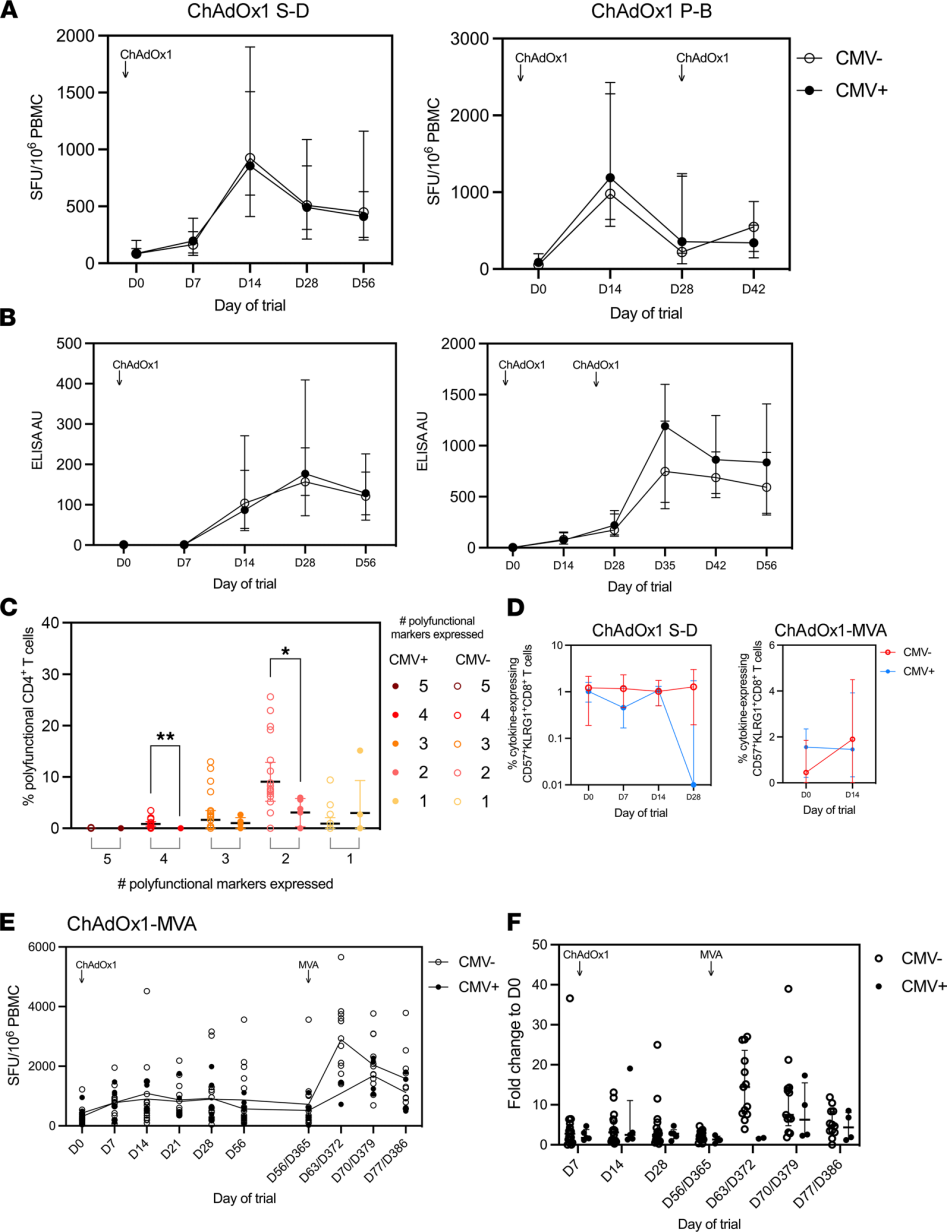
T cell functionality in prime and prime-boost vaccine regimens when stratified by CMV serostatus. (**A**) T cell IFN-γ production from groups 1 and 2 of the ChAdOx1-MVA P-B vaccine regimen measured by ELISPOT and stratified for CMV serostatus. (**B**) tIgG from ChAdOx1 S-D and ChAdOx1 P-B trial participants. (**C**) Polyfunctionality of CD4^+^ T cells from ChAdOx1 S-D participants at day 14 postvaccination, measuring expression of CD25, CD107a, IFN-γ, IL-2, and TNF. (**D**) ChAdOx1 S-D cohort and ChAdOx1-MVA cohort: percentage of cytokine^+^ T cells within the CD8^+^CD57^+^KLRG1^+^ population following vaccination. (**E**) T cell IFN-γ production from groups 1 and 2 of the ChAdOx1-MVA P-B vaccine regimen measured by ELISPOT. (**F**) Fold change of ELISPOT response compared with day 0 from pooled groups 1 and 2 of the ChAdOx2-MVA P-B vaccine regimen and fold change of ELISPOT IFN-γ production when compared with day 0, both stratified for CMV serostatus. ChAdOx1 S-D: *n* = 31 CMV seronegative, *n* = 13 CMV seropositive. ChAdOx1 P-B: *n* = 28 CMV seronegative, *n* = 20 CMV seropositive. ChAdOx1-MVA: *n* = 15 CMV seronegative, *n* = 4 CMV seropositive. Open circles = CMV seronegative, closed circles = CMV seropositive. Statistics conducted using mixed effects analysis with Holm-Šidák multiple comparisons. *P < 0.05, **P < 0.01. Error bars presented as median ± IQR. SFU, spot-forming units (per million cells).

We have previously demonstrated that CMV infection is associated with significantly reduced antigen-specific IgG titers and T cell IFN-γ production after ChAd3-MVA vaccination (37). We investigated how a CMV-correlated reduction in cellular and humoral immunogenicity is affected by alternative prime-boost viral vector vaccination regimens. When stratified by CMV serostatus, there was no significant difference in total T cell IFN-γ when measured by ELISPOT in the ChAdOx1 S-D or ChAdOx1 P-B cohorts following vaccination (Figure 2A). Similarly after either ChAdOx1 S-D or ChAdOx1 P-B regimens, analysis of total IgG antibody titers by ELISA assay did not demonstrate any reduction associated with CMV infection when measured up to day 56 following vaccination (Figure 2B). The T cell ELISPOT and IgG ELISA response were stratified by sex of participant. Within the ChAdOx1 S-D and ChAdOx1 P-B groups, there was no significant difference in the immune response between sexes. Furthermore, there was no significant difference between participant sex and immune response when further stratified by CMV serostatus. Within the ChAdOx1-MVA group, there was an overall significant difference when analyzed using mixed effects analysis on the sex of participants and the ELISPOT response (*P* = 0.044). When stratified further by sex and CMV serostatus, we saw a similar trend toward the loss of T cell ELISPOT responses in CMV-seropositive donors. This response was principally seen in men; however, the group sizes were too small for the difference to reach statistical significance (Supplemental Figure 2; supplemental material available online with this article; https://doi.org/10.1172/jci.insight.154187DS1). However, within the ChAdOx1 S-D cohort, intracellular cytokine analysis of T cell polyfunctionality demonstrated a significant decrease in the frequency of CD4^+^ T cells expressing either 4 or 2 markers of activation (among the 5 markers of CD25, IFN-γ, TNF, CD107a, and IL-2) at day 14 after vaccination in CMV^+^ donors (4 markers, *P* = 0.026; 2 markers, *P* = 0.035; Figure 2C). Within the CD8^+^CD57^+^KLRG1^+^ population, there was no difference in the frequency of total cytokine^+^ T cells following vaccination between CMV serostatus from day 0 to day 28. This was also replicated following ChAdOx1 vaccination in the ChAdOx1-MVA vaccine cohort (Figure 2D).

We also analyzed T cell IFN-γ ELISPOT data from the ChAdOx1-MVA vaccine cohort. These data were stratified for CMV serostatus and groups 1 and 2 were pooled into “postprime” and “postboost” time points. Group 1 received the MVA boost dose at 8 weeks and group 2 at 52 weeks after the boost. Previous analysis shows no significant difference in the T cell IFN-γ response between these 2 groups (29). Although the number of CMV-seropositive donors was low within these groups, analysis of T cell IFN-γ ELISPOT data with regard to CMV serostatus demonstrated a significant association between CMV serostatus and the overall magnitude of the antigen-specific T cell response across time (*P* = 0.0038 time × CMV, fixed effects analysis; Figure 2E). This was also replicated by fold change analysis of ELISPOT response in comparison to day 0 (Figure 2F).

### NK cells have distinct terminally differentiated populations with reduced activation in CMV^+^ participants.

**Figure 3:**
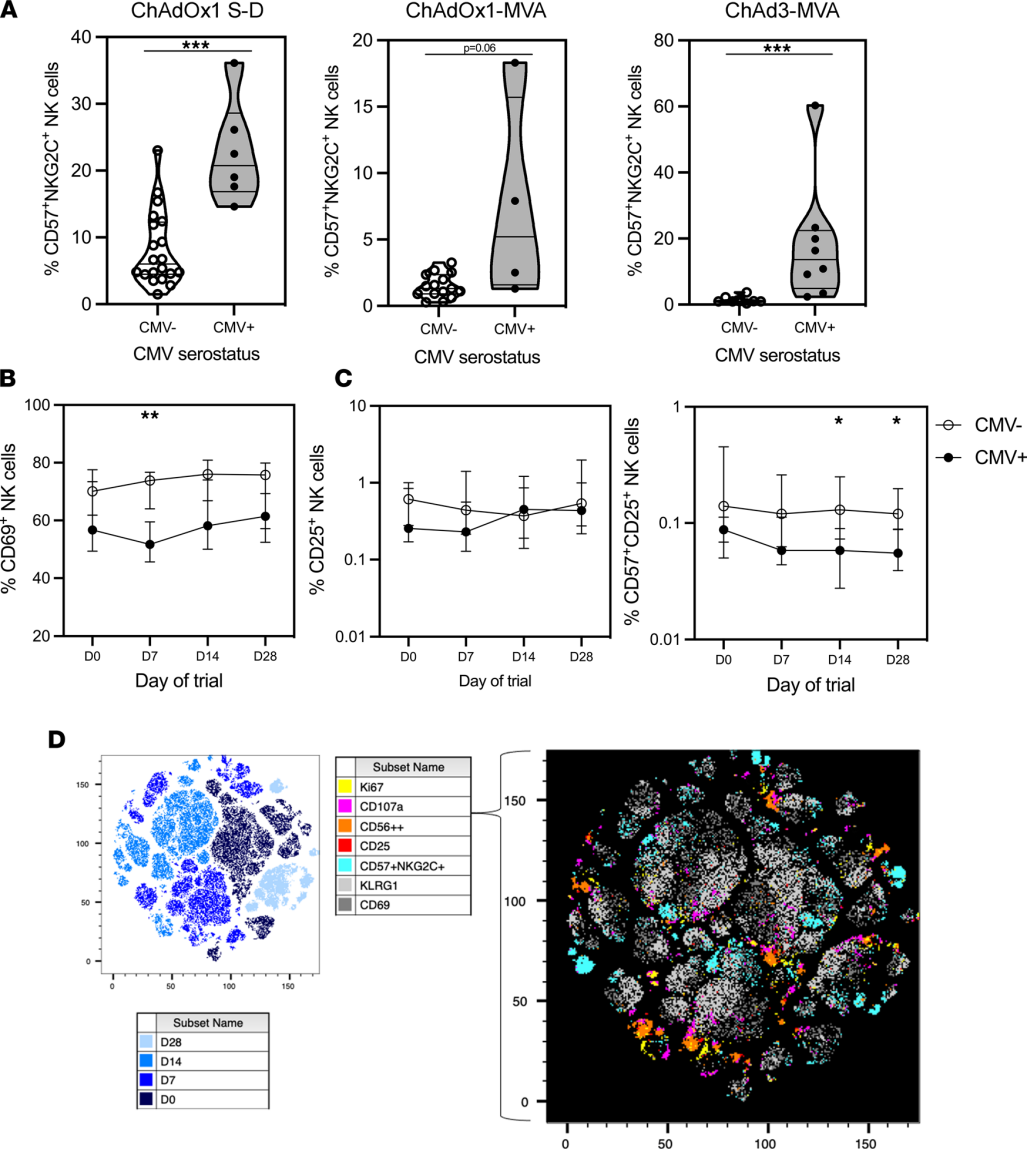
NK cell phenotype with CMV serostatus. (**A**) CD57^+^NKG2C^+^ NK cell frequency with CMV serostatus. (**B**) CD69 expression on NK cells stratified by CMV serostatus. (**C**) Frequency of CD25^+^ NK cells and CD57^+^CD25^+^ NK cells. (**D**) t-SNE analysis conducted on 26 ChAdOx1 cohort samples across 4 time points (day 0, day 7, day 14, and day 28). t-SNE plot was created by downsampling and concatenation of 25,000 randomly selected NK cells from each time point and sample. ChAdOx1 S-D: *n* = 20 CMV seronegative, *n* = 6 CMV seropositive. ChAdOx1-MVA: *n* = 15 CMV seronegative, *n* = 4 CMV seropositive. ChAd3-MVA: *n* = 8 CMV seronegative, *n* = 8 CMV seropositive. Open circles = CMV seronegative, closed circles = CMV seropositive. Statistics conducted using Mann-Whitney *U* test and mixed effects analysis with Holm-Šidák multiple comparisons. *P < 0.05, **P < 0.01, ***P < 0.005. Error bars presented as median ± IQR.

CMV infection has an established impact on the phenotype of NK cells with expansion of a CD57^+^NKG2C^+^ population (38). This was identified in the ChAdOx1 S-D, ChAdOx1-MVA, and ChAd3-MVA vaccination regimens at day 0 (ChAdOx1 S-D, *P* = 0.0003; ChAdOx1-MVA, *P* = 0.06; ChAd3-MVA, *P* = 0.0006; Figure 3A). Further analysis of the ChAdOx1 S-D cohort identified NK cells with a significantly lower expression of CD69 in CMV^+^ participants at day 7 after vaccination (*P* = 0.0011; Figure 3B). Within the ChAdOx1 S-D group, there was no significant reduction in CD25 expression across the total NK cell population; however, there was a significant reduction of CD25 expression within the most mature CD57^+^ NK cell population, which is increased in frequency with CMV seropositivity (39) (days 14 and 28, *P* < 0.03; Figure 3C). t-Distributed stochastic neighbor embedding (t-SNE) analysis was conducted on unstimulated NK cells from the ChAdOx1 S-D cohort across all 4 time points and highlighted regions of phenotypically immature, activated, and proliferating (Ki-67^+^, CD25^+^ CD56^++^, and CD107a^+^) NK cells, which were distinct from terminally differentiated CMV-associated CD57^+^NKG2C^+^ populations. There was also a separate region of CD69^+^KLRG1^+^ NK cells (Figure 3D).

### Total NK cells and NKG2C^+^ NK cells from CMV^+^ trial participants have lower activation and cytokine production after vaccination.

**Figure 4:**
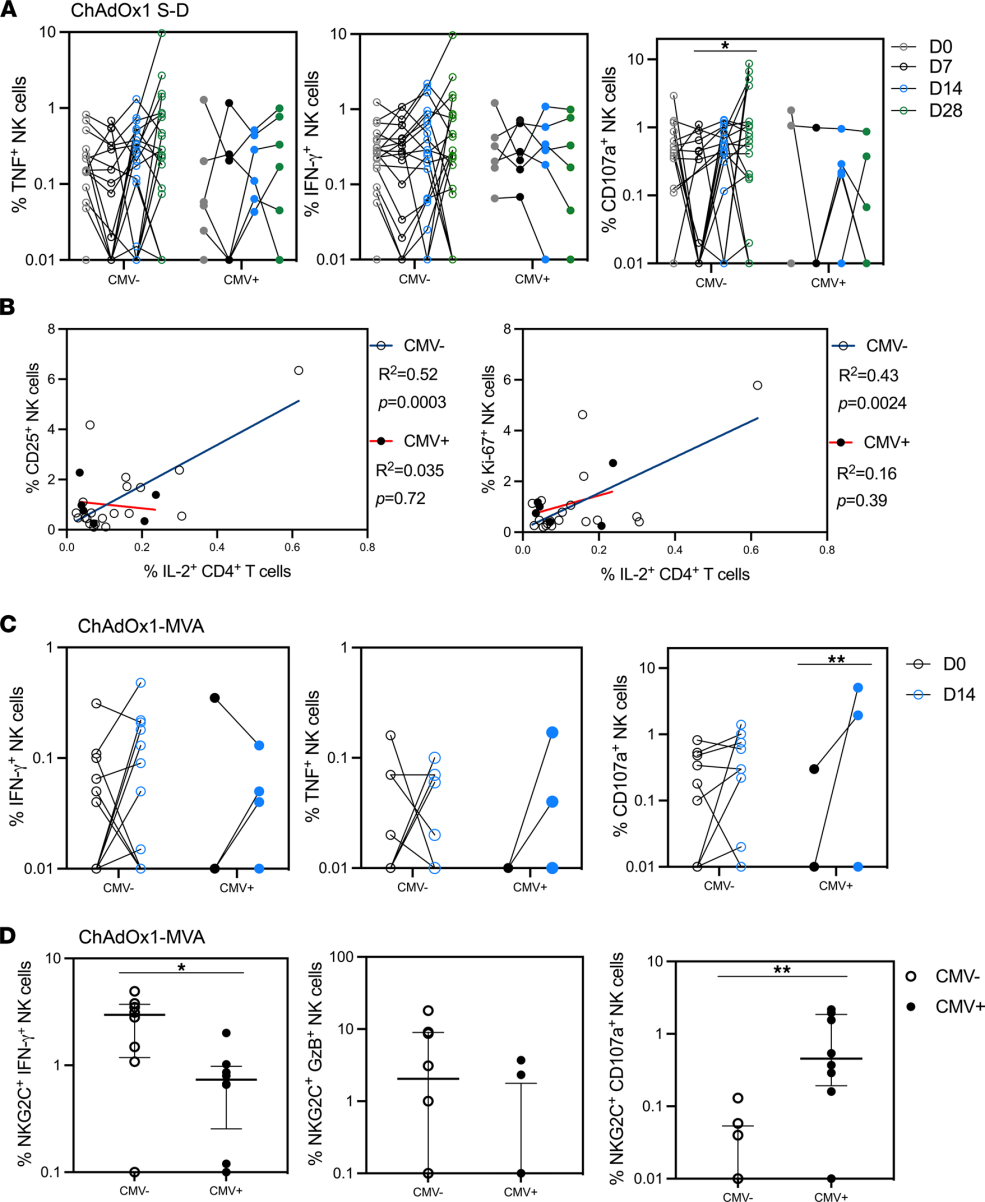
Changes in NK cell cytotoxicity profile with CMV positivity. PBMCs from trial participants stimulated with relevant antigen peptides and stained for cytokine production and cytotoxicity. (**A**) NK cell cytotoxicity and cytokine production (IFN-γ, TNF, and CD107a) from the ChAdOx1 S-D cohort participants. (**B**) Correlation of antigen-specific CD4^+^ IL-2 secretion and NK cell activation (CD25) and proliferation (Ki-67) at day 14 after vaccination stratified by CMV serostatus. (**C**) NK cell cytotoxicity and cytokine production (IFN-γ, TNF, and CD107a) from ChAdOx1-MVA group 1+2 participants. (**D**) ChAd3-MVA participant NKG2C^+^ NK cell cytotoxicity and cytokine production (IFN-γ, granzyme B, and CD107a). ChAdOx1 S-D: *n* = 20 CMV seronegative, *n* = 6 CMV seropositive. ChAdOx1-MVA: *n* = 15 CMV seronegative, *n* = 4 CMV seropositive. ChAd3-MVA: *n* = 8 CMV seronegative, *n* = 8 CMV seropositive. Open circles = CMV seronegative, closed circles = CMV seropositive. Statistics conducted using Mann-Whitney *U* test, linear regression, and mixed effects analysis with Holm-Šidák multiple comparisons. *P < 0.05, **P < 0.01. Error bars shown as median ± IQR.

Within the ChAdOx1 S-D cohort, there was no change in the expression of NK cell IFN-γ or TNF between days 0, 7, 14, and 28 with regard to CMV serostatus following stimulation of PBMCs with SARS-CoV-2 peptides. However, there was a significant increase in CD107a expression between day 7 and day 28 in the CMV-seronegative, but not the CMV-seropositive, cohort (*P* = 0.029; Figure 4A). There was no change in the polyfunctionality of NK cells following peptide stimulation when stratified by CMV serostatus (Supplemental Figure 3A).

Within the ChAdOx1 S-D group, we plotted correlations of NK cell CD25 expression and proliferation (Ki-67) to investigate whether loss of NK cell activation may be associated with the downregulation of other markers of activation; for example, antigen-specific CD4^+^ IL-2 has previously been demonstrated to activate NK cells (40, 41). There was some correlation within the CMV-seronegative cohort between antigen-specific CD4^+^ T cell IL-2 secretion and CD25 expression (goodness of fit *R*^2^ = 0.52, *P* = 0.0003) or Ki-67 expression at 14 days after vaccination (goodness of fit *R*^2^ = 0.43, *P* = 0.0024), but this was nonsignificant in the CMV^+^ cohort (CD25 *R*^2^ = 0.035, *P* = 0.72; Ki-67 *R*^2^ = 0.16, *P* = 0.39) (Figure 4B).

Although the pooled group 1 and group 2 ChAdOx1-MVA cohorts had a smaller sample size, a similar NK cell response to peptide stimulation was seen within the total NK cell population. There was no change in the expression of IFN-γ or TNF after vaccination when associated with CMV serostatus. Unlike the ChAdOx1 S-D cohort, only CMV-seropositive participants had an increase in CD107a expression at day 14 following ChAdOx1 vaccination (*P* = 0.0072), and there was a positive association between CMV infection and NK cell CD107a expression after ChAdOx1 vaccination (fixed effects analysis, *P* = 0.015 when accounting for time point and CMV serostatus; Figure 4C).

In contrast, in the ChAd3-MVA trial group, there was no significant difference between IFN-γ, granzyme B, or CD107a expression within the overall NK cell population between CMV serostatus at day 7 after the MVA boost (Supplemental Figure 3B). However, the NKG2C^+^ NK cell population of CMV-seropositive donors exhibited a differential response to peptide stimulation, producing significantly less IFN-γ (*P* = 0.015), a trend toward less granzyme B (*P* = 0.12), and significantly more CD107a than CMV-seronegative donors (*P* = 0.0044; Figure 4D).

## Discussion

It is essential that vaccines developed against pandemic pathogens provide protection to populations across the globe. Previous research has demonstrated that CMV infection impairs vaccine immunogenicity through a reduction in antigen-specific IgG and T cell IFN-γ induced by influenza and Ebola virus vaccines (6, 10, 37). This effect is especially prevalent in some vaccines trialed in sub-Saharan Africa, where CMV seropositivity is detectable in virtually all participating adults (42) and in nearly 90% of infants (4).

In this paper, we describe the phenotype and functionality of T cells and NK cells following single-dose and heterologous prime-boost viral vector vaccination in participants with and without CMV. CMV-seropositive participants had an expanded CD57^+^KLRG1^+^ and CD45RA^+^ T_EMRA_ profile, and we further demonstrated that CD8^+^ T cells from CMV-seropositive participants had lower expression of the activation marker CD25 (IL-2Rα) and expanded populations of differentiated PD1^+^CD57^+^CD4^+^ T cells. These new profiles further support the evidence that CMV drives an immunosenescent phenotype with reduced activation, but in this study does not affect the antigen-specific T cell effector function or antibody secretion after single-dose ChAdOx1 vaccination.

The T cell immunogenicity following vaccination with either single-dose or homologous-boost ChAdOx1 was not reduced in CMV-seropositive individuals when measured by T cell IFN-γ ELISPOT. This is encouraging for the immunogenicity of ChAdOx1-vectored vaccines in areas of high CMV burden. However, in the ChAdOx1 S-D cohort, there was a significant reduction in the polyfunctionality of CD4^+^ T cells in CMV-seropositive individuals at day 14. Although the sample size for the heterologous ChAdOx1-prime MVA-boost was small, there was a trend toward fewer antigen-specific IFN-γ–secreting Τ cells from CMV-seropositive donor T cells compared with CMV-seronegative donors. This corroborates post-MVA vaccination data from the ChAd3-MVA vaccine trial (37) and suggests that heterologous viral-vectored vaccine regimens that include MVA may drive the association of lower vaccine immunogenicity with CMV serostatus. One hypothesis for why CMV serostatus affects the heterologous prime-boost ChAd-MVA vaccination is that MVA can induce apoptosis in immune cell populations including dendritic cells (DCs), macrophages, and NK cells (43, 44). Apoptosis of MVA-infected DCs has been shown to enhance antigen cross-presentation to CD8^+^ T cells via uninfected DCs and secretion of Th1-polarizing cytokines (43, 44). Potentially, CMV-seropositive donors do not elicit as robust a response due to CMV-mediated downregulation of antigen presentation to CD4^+^ T cells during latent infection (45, 46). This, combined with expanded CMV-specific T cell populations and terminally differentiated T cells that are less responsive to stimulation, may contribute to a loss of antigen-specific vaccine response in CMV-seropositive participants following MVA vaccination. Unlike the ChAd3-MVA trial, we did not see reduced antigen-specific IgG titers with CMV seropositivity in the ChAdOx1 S-D, ChAdOx1 P-B, or ChAdOx1-MVA vaccine cohorts. This suggests that vaccine immunogenicity in low- to middle-income countries, where CMV seropositivity is high, will be equal to immunogenicity in UK trials.

The NK cell response to vaccination was more heterogeneous than the T cell response. Within the CMV-seropositive donors, we identified a marked reduction of total CD69 expression and CD25 (IL-2Rα) expression on CD57^+^ NK cells. Other research has previously demonstrated that CD57^+^ NK cells are expanded during CMV infection and that CD57^+^ NK cells have epigenetic modulation of the *IFNG* locus (47) and reduced expression of cytokine receptors for IL-2, IL-12, IL-15, and IL-18 (48, 49).

NK cell activation following vaccination and infection can be modulated by antigen-specific CD4^+^ T cell IL-2 secretion (17, 40, 50, 51). We demonstrate a positive correlation between CD4^+^ T cell IL-2 secretion and total NK cell activation and proliferation, measured by CD25 and Ki-67, respectively, in ChAdOx1 S-D CMV-seronegative participants but not in CMV-seropositive participants. Loss of NK cell cytotoxicity to pertussis vaccination has previously been demonstrated to correlate with increased frequency of CD57^+^ NK cells (48); thus, expansion of the CD57^+^ NK cell population in CMV-seropositive donors may affect total NK cell activation to IL-2 secretion. However, further studies are needed to demonstrate if this phenomenon underpins the observations measured here.

NK cell cytokine secretion varied between vaccine cohorts. In the ChAdOx1 S-D cohort, we observed more CD107a expression in the CMV-seronegative participants following vaccination; however, in the ChAdOx1-MVA and ChAd3-MVA cohorts, CMV-seropositive individuals expressed more CD107a after vaccination. Previous studies have found either no effect of CMV infection on NK cell CD107a expression (18, 52) or reduced expression with CMV seropositivity (19). Increased expression of CD107a in our cohorts may be due to a proportional increase in CD57^+^ NK cells in CMV^+^ participants, which are less reactive to activation by cytokines and cytokine secretion but are still able to degranulate following stimulation (48). NK cells from CMV-seropositive donors may also exert a regulatory function on T cells through increased degranulation and IL-10 secretion, as previously demonstrated during murine CMV infection (53, 54). It is currently unknown whether these phenotypic and functional differences in NK cells have an impact on overall vaccine efficacy.

This study was limited to healthy UK adults aged 18–55 who were enrolled in clinical vaccine trials through the University of Oxford. Analysis was conducted on vaccines with different antigen inserts due to sample availability and ongoing clinical trials. Therefore, we cannot rule out a potential effect of the encoded vaccine antigen that may confound these data. The average age of ChAdOx1 S-D, ChAdOx1 P-B, ChAdOx1-MVA, and ChAd3-MVA volunteers was 33 (range: 18–54), 39 (range: 19–55), 25 (range: 19–46), and 33 years (range: 21–50), respectively, thus limiting the possibility of stratifying by age across trials. Due to the opportunistic nature of sample collection, CMV serotyping was also conducted retrospectively after analysis, preventing even distribution of CMV serostatus and cohort size and limiting sample size to the recruitment of the trial group.

We have now demonstrated across 3 viral-vector vaccine trials against different pathogens that CMV contributes toward an immunosenescent change in T cell and NK cell phenotype and a reduction in effector function by cytotoxic leukocytes in healthy UK adults. However, these data suggest that the negative correlation between CMV IgG titers and vaccine immunogenicity may be less prevalent in single-dose or homologous-boost ChAdOx1 vaccine regimens in UK adults and exacerbated following heterologous vaccination with MVA. Additional investigation will be required in other demographics with a higher burden of CMV and endemic pathogens to further elucidate the interaction of CMV infection and vaccination. Although we cannot determine if CMV infection affects vaccine efficacy from these data, we would speculate that efficacy will not be affected following single-dose or homologous-boost ChAdOx1 vaccination as the overall humoral and cellular responses do not differ between CMV^+^ and CMV^–^ cohorts. These results, therefore, are encouraging for ongoing global vaccination efforts and indicate that homologous boosting with ChAdOx1 as used in the vaccine regimen may not be significantly affected by CMV-driven loss of immunogenicity.

## Methods

### Clinical trials

#### ChAd3-MVA (EBL04).

The EBL04 clinical trial (NCT02485912) was a phase Ia trial conducted in Oxford, United Kingdom, in healthy adults aged 18–50 years old. This trial used the replication-deficient ChAd3 encoding the glycoprotein from EBO-Z administered at a dose of 1 × 10^8^ PFU and the replication of deficient MVA-expressing EBO-Z glycoprotein at a dose of 1.5 × 10^8^ PFU (31). Group 2 (*n* = 16, *n* = 8 CMV^+^ and *n* = 8 CMV^–^) included in this manuscript had a prime-boost regimen with an interval of 1 week. All clinically available data were included, and demographic data can be found in the original publication (31).

#### ChAdOx1-MVA (FLU005).

The FLU005 clinical trial (NCT01818362) was a phase I trial conducted in Oxford, United Kingdom, in healthy adults aged 18–50 using used replication-deficient ChAdOx1 encoding the NP and M1 as a fused protein from influenza A H3N2/A/Panama/2007/99 at a dose of 2.5 × 10^10^ v.p. and MVA encoding NP+M1 at a dose of 1.5 × 10^8^ PFU (29). Groups 1 and 2 (*n* = 19, *n* = 15 CMV^–^, *n* = 4 CMV^+^) included in this manuscript had a prime-boost interval of 8 weeks and 52 weeks, respectively. All clinically available data were included, and demographic data can be found in the original publication (29).

#### ChAdOx1/ChAdOx1 P-B (COV001/COV002).

The COV001 phase I clinical trial (NCT04324606) was conducted in Oxford, United Kingdom, in healthy adult volunteers aged 18–55 (group 1, *n* = 44, *n* = 31 CMV^–^, *n* = 13 CMV^+^). Volunteers were vaccinated with 1 dose of ChAdOx1 encoding the spike glycoprotein from SARS-CoV-2 (nCoV-19) at a dose of 5 × 10^10^ v.p.

The COV002 clinical trial (NCT04400838) was conducted on healthy UK volunteers aged 18–55 (group 5d, *n* = 27, *n* = 14 CMV^+^, *n* = 13 CMV^–^) who were administered 2 doses of ChAdOx1 nCoV-19 at a dose of 5 × 10^10^ v.p. and an interval of 28 days. Control group vaccine participants (none included in this analysis) were administered the MenACWY vaccine (35, 36, 55, 56). Cytometry analysis conducted on group 1 participants: *n* = 26 (*n* = 20 CMV^–^, *n* = 6 CMV^+^). All clinically available data were included, and demographic data can be found in the original publications (35, 36, 55, 56).

### T cell ELISPOT and total IgG ELISA

T cell IFN-γ ELISPOT responses were assessed ex vivo using fresh PBMC as previously described for each clinical trial (29, 31, 35). Total antigen-specific IgG ELISA responses were calculated from standardized ELISA assays developed for each trial (31, 35, 36).

### CMV serotyping

CMV seroprevalence was assessed in day 0 samples from clinical trial plasma. Anti-CMV IgG ELISA kits were used following manufacturer’s instructions (Abcam, ab108724). Briefly, serum was diluted 1:100 in dilution buffer, and 100 μL was plated in duplicate on ELISA plates coated with CMV antigens. Positive, negative, and cutoff controls were also included. Plates were incubated at 37°C for 1 hour in the dark and then washed 3 times with 300 μL wash buffer. Wells were then incubated in the dark at room temperature with 100 μL CMV anti-IgG HRP conjugate from the kit for 30 minutes. Plates were developed with 100 μL TMB substrate solution for 15 minutes in the dark. The reaction was stopped with the addition of 100 μL stop solution. Plates were read at 405 nm within half an hour of development. Standardized ELISA units were calculated as (OD value × 10)/cutoff value.

PBMC defrosting and stimulation

Vials with 1 mL of PBMCs from vaccine trial donors were selected from –180°C or liquid nitrogen storage. Cells were kept on dry ice until defrosting. Vials were defrosted in 37°C water bath and transferred into 9 mL prewarmed complete RPMI medium (Gibco) supplemented with FCS (Gibco), l-glutamine, and penicillin/streptomycin (R10) with 2 U/mL Benzonase (all from MilliporeSigma) and incubated for up to 2 hours. Cells were then centrifuged at 400*g* at room temperature for 5 minutes and resuspended in 10 mL of fresh R10. Cells were counted using trypan blue or a Casy counter and resuspended in a concentration of 2 × 10^7^ cells/mL.

Then, 1 × 10^6^ to 2 × 10^6^ PBMCs per well were plated in a 96-well plate and stimulated with a final concentration of 1–2 μg/mL synthetic peptides. A total of 100 μL media was used as a negative control, and 0.1 μL/well PMA-ionomycin was used as a positive control. For T cell analysis, PBMCs were costimulated in the presence of 0.2 μL/well anti-CD28 and anti-CD49d (Life Technologies). For T cell and NK cell analysis, cells were also incubated with anti-CD107a (Supplemental Table 1). Cells were incubated for 2 hours at 37°C, then incubated for a further 16 hours following the addition of 0.1 μL/well Brefeldin A and Monensin (BioLegend).

### Flow cytometry

The panel and method for lymphocyte staining on the Cytek Aurora cytometer have been previously published (27). Briefly, PBMCs were centrifuged at 400*g* for 3 minutes, supernatant was discarded, and cells were resuspended and washed in 200 μL FACS buffer (Gibco, Dulbecco’s PBS + 5% BSA). After removal of supernatant, cells were resuspended in 100 μL of surface cocktail antibody stain (Supplemental Table 1) and incubated in the dark for 30 minutes at 4°C. A total of 100 μL FACS buffer was added, and cells were washed twice by centrifugation at 400*g* for 5 minutes at room temperature and by discarding supernatant. For intracellular staining, PBMCs were incubated in CytoFix/CytoPerm solution (BD Biosciences) for 30 minutes in the dark at 4°C. Cells were washed twice in 100–200 μL Perm/Wash buffer and incubated for 30 minutes in the dark at 4°C in 100 μL of intracellular antibody cocktail (Supplemental Table 1). Cells were then washed twice in Perm/Wash and once in FACS buffer and resuspended in 100–200 μL FACS buffer for acquisition on the BD LSRFortessa using FACSDiva (BD Biosciences) or Cytek Aurora using SpectraFlo (Cytek Biosciences). Single-fluorochrome compensation was calculated from single-stained beads (BD Biosciences) or human PBMCs. Data analysis was conducted by hierarchical gating in FlowJo v10.7.1 and Prism 8 (GraphPad) (Supplemental Figure 1). Peptide-specific responses were calculated by subtracting unstimulated sample data from stimulated sample data.

### Statistics

Normality testing of data was conducted using the Shapiro-Wilk test or the D’Agostino-Pearson test. For non-normally distributed data or small sample groups, analysis on single time point data was conducted using the Mann-Whitney *U* test. Across multiple time points, mixed effects analysis with Holm-Šidák multiple comparisons was used. All data analysis was conducted in GraphPad Prism v8. *P* values, where appropriate, are given within the text and figure legends. Data are presented as median ± IQR. Statistical significance was defined as *P < 0.05, **P < 0.01, ***P < 0.005, and ****P < 0.0001. A *P* value of less than 0.05 was considered significant.

The t-SNE plot analysis was conducted by downsampling in FlowJo v 10.7.1. A random sample of 25,000 live NK cells were collected per donor and time point and concatenated into a single file. Relevant NK cell markers (CD56, CD57, CD16, NKG2C, CD107a, Ki-67, CD27), time point, and CMV serostatus were included as parameters. The t-SNE analysis was implemented with 1000 iterations and a perplexity of 30 using the Barnes-Hut gradient algorithm.

### Study approval

Participants provided written informed consent before inclusion in these trials. Trials were conducted according to the principles of the Declaration of Helsinki. The EBL04 clinical trial (ClinicalTrials.gov: NCT02485912) was reviewed and approved by the UK National Research Ethics Service (committee South Central — Oxford A, ref: 15/SC/0108) and the Medicines and Healthcare Products Regulatory Agency (ref: 21584/0341/001-0001). The COV001 clinical trial (ClinicalTrials.gov: NCT04324606) was approved in the United Kingdom by the Medicines and Healthcare Products Regulatory Agency (ref: 21584/0424/001-0001) and the South Central Berkshire Research Ethics Committee (ref: 20/SC/0145). The COV002 clinical trial (ClinicalTrials.gov: NCT04400838) was approved in the United Kingdom by the Medicines and Healthcare Products Regulatory Agency (ref: 21584/0428/001-0001) and the South Central Berkshire Research Ethics Committee (ref: 20/SC/0179).

## Author contributions

HRS and TL conceived and designed the study and TL is the principal investigator. HRS, TL, SG, and PK contributed to the design of the study. HRS, NMP, and GSB were responsible for laboratory testing, assay development, and data collection. PMF, SBR, AF, RM, KJE, AJP, and AVSH contributed to the implementation of the study and/or data collection. HRS, NMP, PK, SG, AJP, and TL contributed to the preparation of the report. All authors critically reviewed and approved the final version.

## Supplementary Material

Supplemental data

Supplemental table 1

## Figures and Tables

**Table 1:**
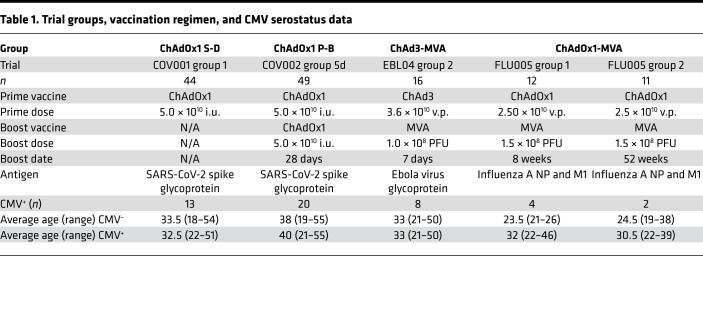
Trial groups, vaccination regimen, and CMV serostatus data

## Version 1 02/22/2022

In-Press Preview

## Version 2 03/22/2022

Electronic publication
