# Supplementary material for: CMV-associated T cell and NK cell terminal differentiation does not affect immunogenicity of ChAdOx1 vaccination
Source: JCI Insight. 2022 Mar 22;7(6):e154187. doi: 10.1172/jci.insight.154187 (PMC8986084; doi:10.1172/jci.insight.154187)
Supplement: Supplemental data [file jciinsight-7-154187-s093.pdf]

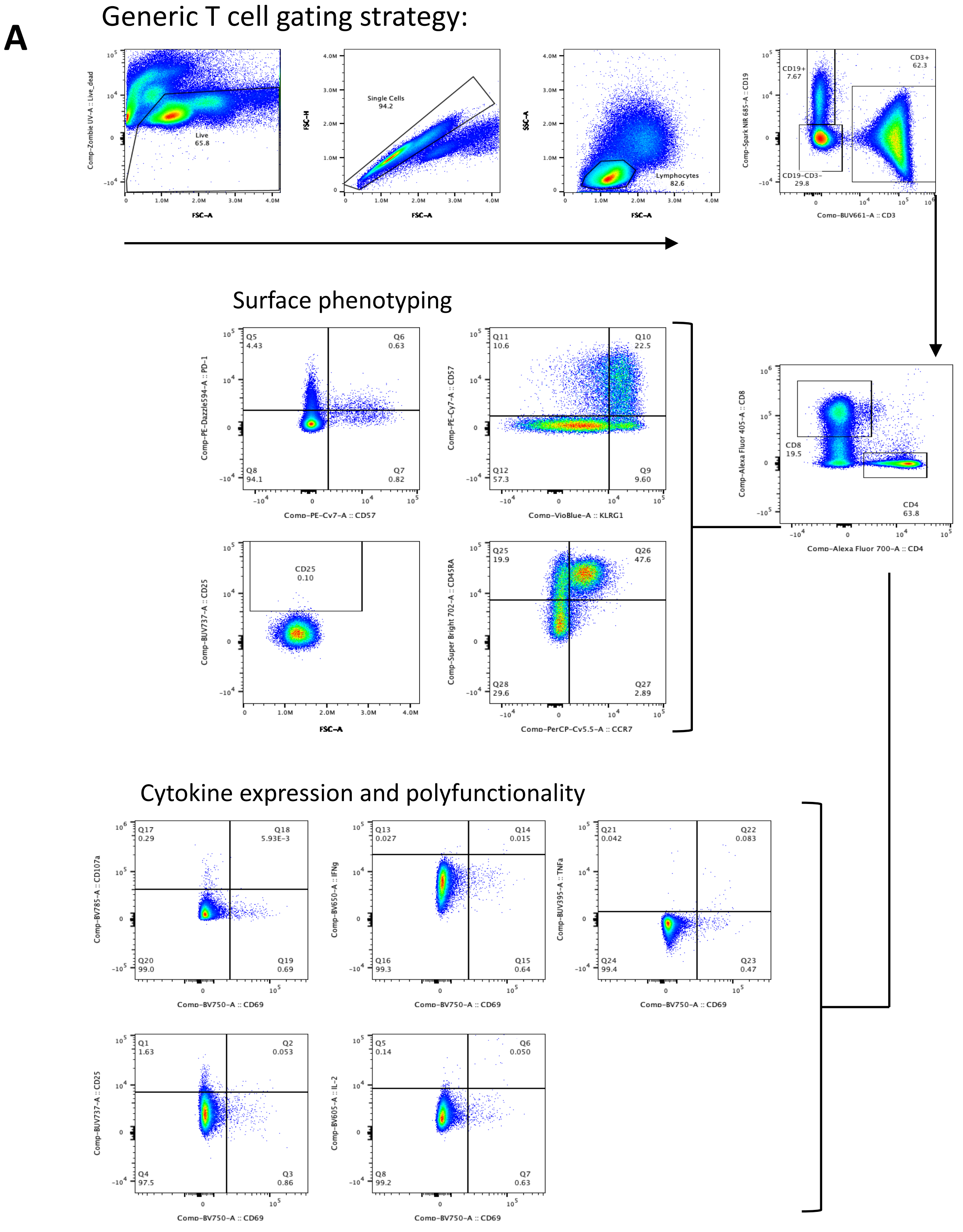

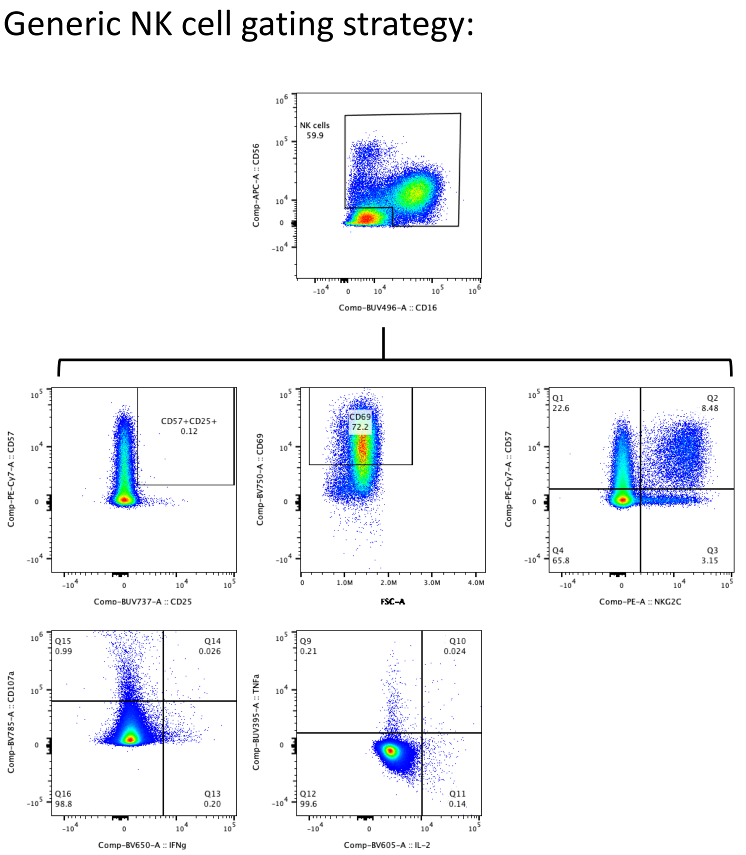

Figure S1: Gating strategy for T cells and NK cells A) T cells were gated in the order shown. Surface phenotype analysis conducted on CD45RA/CCR7 memory populations, CD57/KLRG1 expression, CD57/PD1 expression, and CD25 expression. Cytokine expression and polyfunctionality analysis conducted on CD69^{+} T cells expressing IFN-g, TNF, CD107a, IL-2 and CD25. B) NK cells were gated as shown. Surface phenotype analysis conducted on CD25/CD57 expression, CD69 expression, and CD57/NKG2C expression. Cytokine analysis conducted on CD107a/IFNg and TNF/IL-2 populations.

FSC-A = forward scatter area, SSC-A = side scatter area, SSC-H = side scatter height.

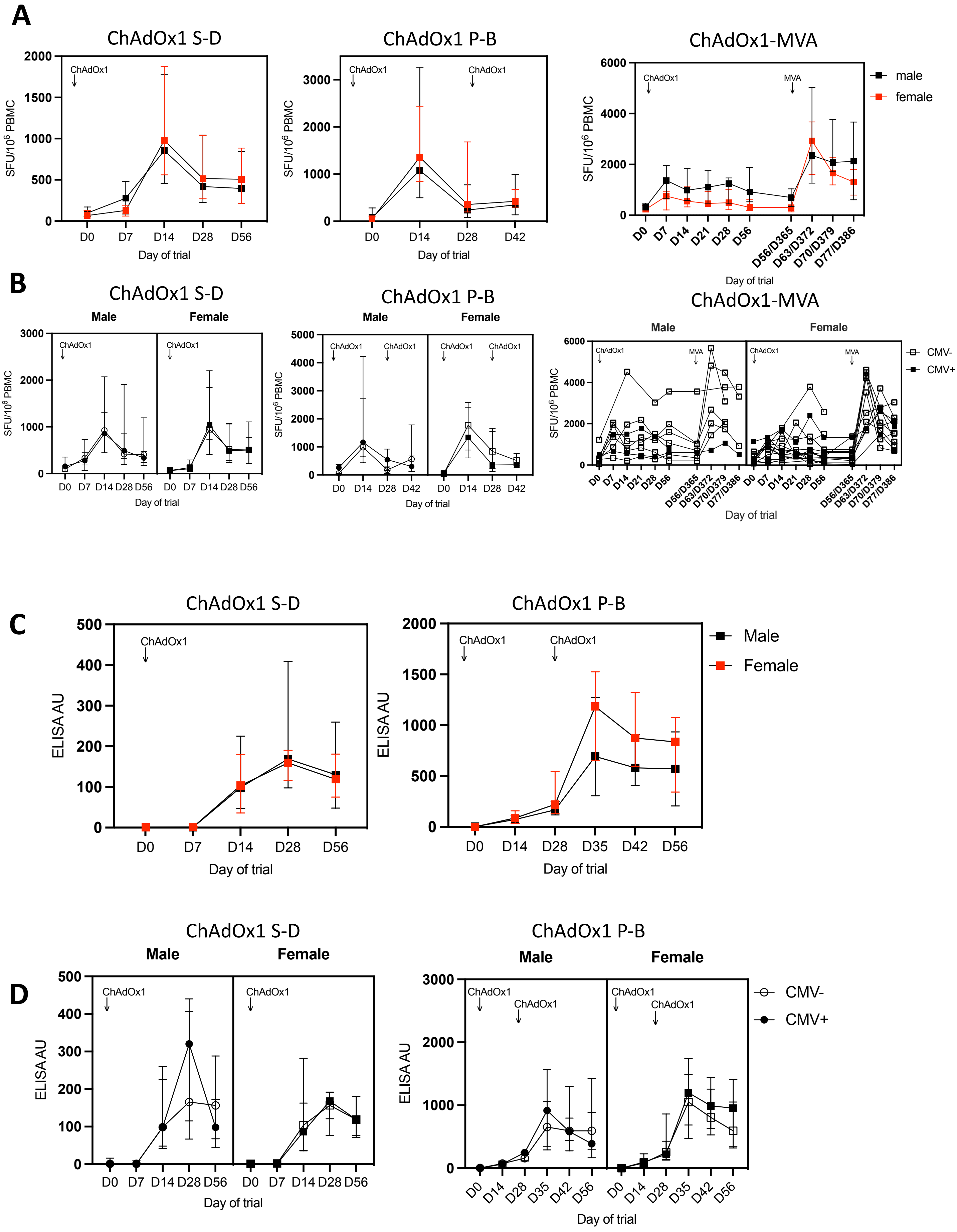

Figure S2. T cell ELISpot and IgG ELISA responses stratified by participant sex and CMV serostatus

T cell IFN-g production from ChAdOx1 S-D, ChAdOx1 P-B, and ChAdOx1-MVA vaccine trial groups measured by ELISpot. A) ELISpot responses stratified by sex of participant, B) ELISpot responses stratified by sex of participant and CMV serostatus.

IgG responses from ChAdOx1 S-D and ChAdOx1 P-B vaccine trial groups measured by ELISA. C) ELISA responses stratified by sex of participant. D) ELISA responses stratified by sex of participant and CMV serostatus.

Statistics conducted using mixed-effect analysis with Sidak multiple comparisons. Error bars shown as median± IQR.

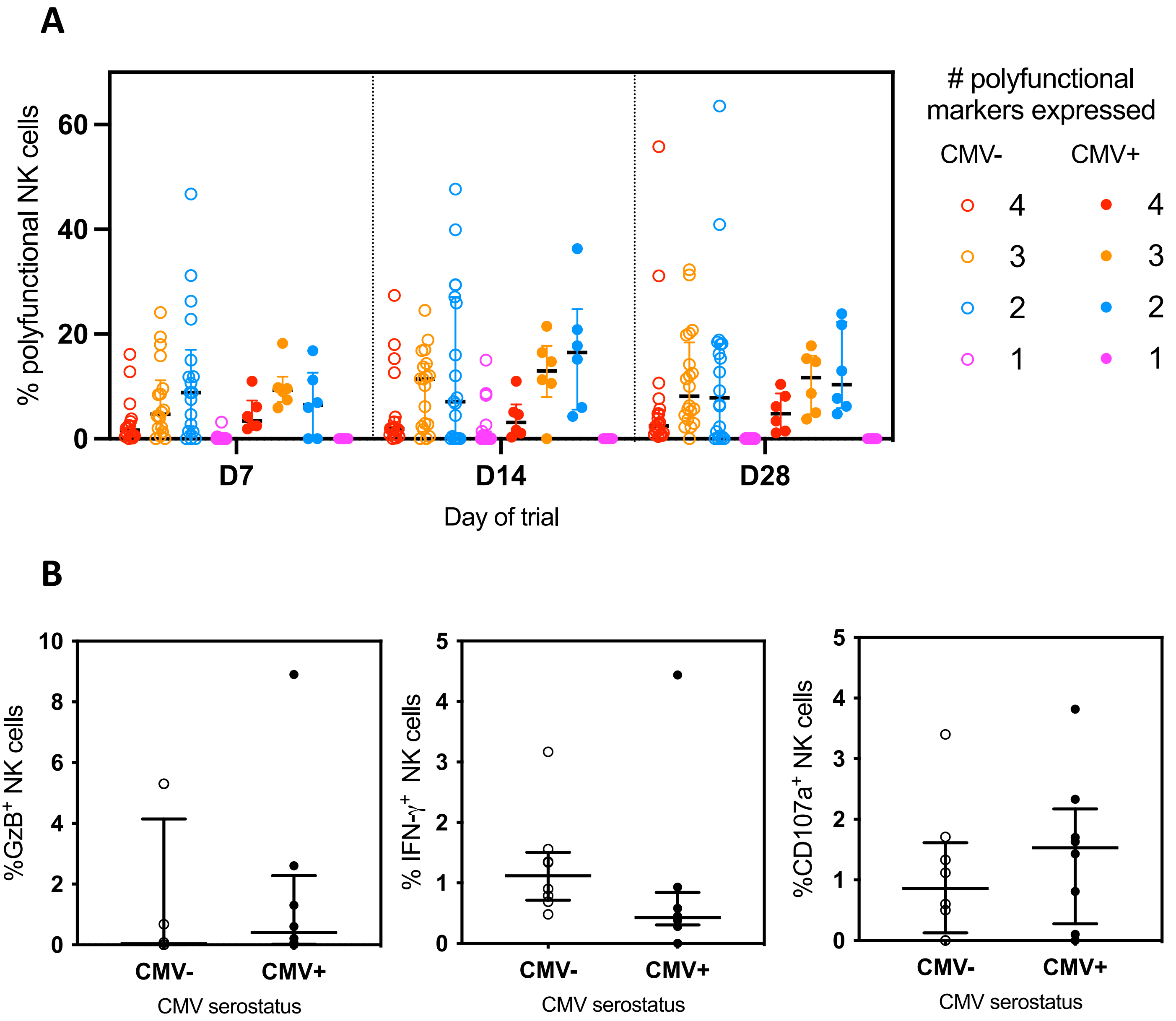

Figure S3. Non-significant changes in NK cell functional response

PBMCs from trial participants were stimulated with relevant antigen peptides and stained for cytokine production and cytotoxicity. A) NK cell polyfunctional response (measured by IFN-g, IL-2 TNF and CD107a) from the ChAdOx1 S-D cohort participants. B) Total NK cell population production of CD107a, IFN-g and granzyme B from the ChAd3-MVA cohort. ChAdOx1 S-D n=20 CMV seronegative, n=6 CMV seropositive. ChAd3-MVA n=8 CMV seronegative, n=8 CMV seropositive. Open circles = CMV seronegative, closed circles = CMV seropositive.

Statistics conducted using Mann-Whitney U test and mixed-effect analysis with Sidak multiple comparisons *$p<0.05$, **$p<0.01$, ***$p<0.005$. Error bars shown as median± IQR.
