# Supplementary material for: CMV-associated T cell and NK cell terminal differentiation does not affect immunogenicity of ChAdOx1 vaccination
Source: JCI Insight. 2022 Mar 22;7(6):e154187. doi: 10.1172/jci.insight.154187 (PMC8986084; doi:10.1172/jci.insight.154187)
Supplement: Supplemental table 1 [file jciinsight-7-154187-s094.pdf]

Table S1. Human lymphocyte antibodies for Cytek Aurora and BD LSR-II cytometers				
**Antibody**	**Fluorochrome**	**Clone**	**Supplier**	**Titration**
Live/Dead	Zombie UV	N/A	BioLegend	1 in 250
CD3	BUV661	OKT3	ThermoFisher Scientific, UK	1 in 400
CD4	AF700	OKT4	BioLegend	1 in 400
CD8	AF405	3B5	ThermoFisher Scientific, UK	1 in 100
CD19	Spark NIR 685	CD19.11	BioLegend	1 in 200
CD56	APC	5.1H11	BioLegend	1 in 400
CD16	BUV496	3G8	BD BioSciences	1 in 200
CD45RA	Superbright 702	HI100	ThermoFisher Scientific, UK	1 in 100
CCR7	PerCP Cy5.5	G043H7	BioLegend	1 in 50
PD1	PE Dazzle 594	EH12.2H7	BioLegend	1 in 100
CD57	PE Cy7	HNK-1	BioLegend	1 in 100
CD27	PerCP eF710	O323	ThermoFisher Scientific, UK	1 in 200
KLRG1	VioBlue	2F1/KLRG1	Miltenyi	1 in 100
CD138	BUV805	MI25	BD Biosciences	1 in 100
NKG2C	PE	REA205	Miltenyi	1 in 200
NKG2A	BV480	131411	BD BioSciences	1 in 100
CD71	BUV563	L01.1	BD Biosciences	1 in 100
CD20	AF532	2H7	ThermoFisher Scientific, UK	1 in 50
IgM	BB515	G20-127	BD BioSciences	1 in 100
CD25	BV737	M-A251	BioLegend	1 in 100
CD69	BV750	FN50	BD BioSciences	1 in 100
IgG	BV650	G18-145	BD BioSciences	1 in 100
Ki-67	APC eF780	SolA15	ThermoFisher Scientific, UK	1 in 100
IFN-γ	BV650	42.B3	BioLegend	1 in 50
CD107a	BV786	H4A3	BioLegend	1 in 100
TNFα	BUV395	Mab11	BD BioSciences	1 in 100
IL-2	BV605	MQ1-17H12	BioLegend	1 in 100
CD14	eF506	61D3	ThermoFisher Scientific, UK	1 in 100
CD19	eF506	HIB19	ThermoFisher Scientific, UK	1 in 100
CD3	eF506	UCHT1	BioLegend, UK	1 in 100
Live/dead	Aqua	N/A	ThermoFisher Scientific, UK	1 in 1000
CD56	PE or PerCP/Cy5.5	HCD56	BioLegend, UK	1 in 100 or 1 in 33
CD16	BV711	3G8	BioLegend, UK	1 in 100
CD57	PE	HCD57	BioLegend, UK	1 in 50
NKG2A	APC	REA110	Miltenyi	1 in 50
NKG2C	PE or PE-vio770	REA205	Miltenyi	1 in 33
IFN-γ	FITC	4S.B3	BioLegend, UK	1 in 200
GzB	AF700	QA16A02	BioLegend, UK	1 in 50
CD107a	BV421	H4A3	BioLegend, UK	1 in 500
